# Nogo-A Is Critical for Pro-Inflammatory Gene Regulation in Myocytes and Macrophages

**DOI:** 10.3390/cells10020282

**Published:** 2021-01-31

**Authors:** H. M. Arif Ullah, A. K. Elfadl, SunYoung Park, Yong Deuk Kim, Myung-Jin Chung, Ji-Yoon Son, Hyun-Ho Yun, Jae-Min Park, Jae-Hyuk Yim, Seung-Jun Jung, Young-Chul Choi, Jin-Hong Shin, Dae-Seong Kim, Jin-Kyu Park, Kyu-Shik Jeong

**Affiliations:** 1 Department of Pathology, College of Veterinary Medicine, Kyungpook National University, Daegu 41566, Korea; arif55dml@knu.ac.kr (H.M.A.U.); ahmedpath@hotmail.com (A.K.E.); sunnypark78@gmail.com (S.P.); jin6850@naver.com (M.-J.C.); jiyoon1095@naver.com (J.-Y.S.); dbsgusgh7154@naver.com (H.-H.Y.); kow612@naver.com (J.-M.P.); nice7676@naver.com (J.-H.Y.); jsj2845@naver.com (S.-J.J.); jinkyu820@knu.ac.kr (J.-K.P.); 2 Stem Cell Therapeutic Research Institute, Kyungpook National University, Daegu 41566, Korea; 3 Laboratory of Physiology and Cell Signaling, College of Veterinary Medicine, Kyungpook National University, Daegu 41566, Korea; 4 Department of Pathology, Faculty of Veterinary Medicine, University of Khartoum, Khartoum 13314, Sudan; 5 School of Applied Biosciences, Kyungpook National University, Daegu 41566, Korea; ydkim94@knu.ac.kr; 6 Department of Neurology, Gangnam Severance Hospital, Yonsei University College of Medicine, Seoul 03722, Korea; ycchol@yuhs.ac.kr; 7 Department of Neurology, Pusan National University Yangsan hospital, Yangsan 50612, Korea; shinzh@gmail.com (J.-H.S.); dskim@pusan.ac.kr (D.-S.K.)

**Keywords:** Nogo-A, inflammation, CHOP, macrophages, pro-inflammatory factors

## Abstract

Nogo-A (Rtn 4A), a member of the reticulon 4 (Rtn4) protein family, is a neurite outgrowth inhibitor protein that is primarily expressed in the central nervous system (CNS). However, previous studies revealed that Nogo-A was upregulated in skeletal muscles of Amyotrophic lateral sclerosis (ALS) patients. Additionally, experiments showed that endoplasmic reticulum (ER) stress marker, C/EBP homologous protein (CHOP), was upregulated in gastrocnemius muscle of a murine model of ALS. We therefore hypothesized that Nogo-A might relate to skeletal muscle diseases. According to our knocking down and overexpression results in muscle cell line (C2C12), we have found that upregulation of Nogo-A resulted in upregulation of CHOP, pro-inflammatory cytokines such as interleukin (IL)-6 and tumor necrosis factor (TNF)-α, while downregulation of Nogo-A led to downregulation of CHOP, IL-6 and TNF-α. Immunofluorescence results showed that Nogo-A and CHOP were expressed by myofibers as well as tissue macrophages. Since resident macrophages share similar functions as bone marrow-derived macrophages (BMDM), we therefore, isolated macrophages from bone marrow to study the role of Nogo-A in activation of these cells. Lipopolysaccharide (LPS)-stimulated BMDM in Nogo-KO mice showed low mRNA expression of CHOP, IL-6 and TNF-α compared to BMDM in wild type (WT) mice. Interestingly, Nogo knockout (KO) BMDM exhibited lower migratory activity and phagocytic ability compared with WT BMDM after LPS treatment. In addition, mice experiments data revealed that upregulation of Nogo-A in notexin- and tunicamycin-treated muscles was associated with upregulation of CHOP, IL-6 and TNF-α in WT group, while in Nogo-KO group resulted in low expression level of CHOP, IL-6 and TNF-α. Furthermore, upregulation of Nogo-A in dystrophin-deficient (mdx) murine model, myopathy and Duchenne muscle dystrophy (DMD) clinical biopsies was associated with upregulation of CHOP, IL-6 and TNF-α. To the best of our knowledge, this is the first study to demonstrate Nogo-A as a regulator of inflammation in diseased muscle and bone marrow macrophages and that deletion of Nogo-A alleviates muscle inflammation and it can be utilized as a therapeutic target for improving muscle diseases.

## 1. Introduction

Inflammation is the host’s fundamental protective biological response to harmful stimuli, which include infections and tissue damage [1,2,3]. Dysregulated inflammatory responses contribute to the pathophysiology of many chronic diseases [1], and excessive inflammatory responses can damage muscle fibers, which can lead to muscle fibrosis, delays in tissue regeneration, and chronic muscle injury [4,5]. Pro-inflammatory factors are crucial factors in muscle disorders, such as Duchenne muscular dystrophy (DMD), which is a progressive form of muscular dystrophy [6]. Tissue-resident macrophages play an essential role in tissue homeostasis and in the resolution of inflammation [7].

Macrophages are innate immune cells that can differentiate into different phenotypes in response to environmental cues. The two canonical types of macrophages are pro-inflammatory (M1 macrophages) and anti-inflammatory (M2 macrophages) [8,9]. M1 macrophages secrete pro-inflammatory cytokines, chemokines, and enzymes, such as interleukin (IL)-6, IL-1β, tumor necrosis factor (TNF)-α, nuclear factor (NF)-κB, chemokine (C-X-C motif) ligand 1 (CXCL1), chemokine (C-X-C motif) ligand 2 (CXCL2), and inducible nitric oxide synthase (iNOS), which are important for multiple inflammatory processes [1,8,10]. In contrast, M2 macrophages produce anti-inflammatory factors, such as IL-10, cluster of differentiation (CD)-206, and arginase (ARG)-1, which are involved in the resolution of inflammatory processes and the mediation of wound healing [1,8]. Due to the diverse functions of macrophages in controlling immune responses and metabolism, dysregulation of macrophage polarization is associated with disease [11]. Notexin is a myotoxic agent, and lipopolysaccharide (LPS), which is a component of bacterial endotoxin, is involved in severe inflammation by stimulating various pro-inflammatory factors [12,13,14]. However, the molecular mechanisms through which Nogo-A induces inflammation remain unknown.

As a major site of protein folding, the endoplasmic reticulum (ER) is an important cellular organelle [15,16,17]. ER stress occurs when unfolded or misfolded proteins accumulate in the ER [16,18]. Three protein sensors are located at the ER membrane, where they activate transcription factor (ATF)-6, inositol requiring enzyme (IRE)-1α, and PKR-like ER kinase (PERK), which function in the identification of increased ER stress and subsequently activate the unfolded protein response (UPR) [14,19,20]. Activation of the UPR results in activation and upregulation of C/EBP homologous protein (CHOP) [21,22], which is a transcription factor that indicates ER stress [23]. ER stress is an essential cellular response that triggers inflammation [14,16,18]. Several studies have revealed that ER stress is involved in various pathophysiological conditions including autoimmune diseases, inflammatory diseases, neurodegenerative diseases, cancer, and metabolic diseases [14,23,24].

Nogo-A is known as a neurite outgrowth inhibitor. It is a member of the reticulon 4 (Rtn4) family of proteins, is localized within the ER membrane, and is essential for the regulation of the tubular structure of the ER [25,26,27]. Nogo has three splicing isoforms Nogo-A (Rtn 4A), Nogo-B (Rtn 4B), and Nogo-C (Rtn 4C), and these isoforms contain the same carboxy terminal but different amino terminals [27,28,29]. Nogo-A (200 kDa) is a high-molecular-weight membrane protein that is primarily expressed in the central nervous system (CNS). Nogo-A acts as a growth inhibitory factor [30,31,32] that influences the migration of cells in the neural tube and is a key negative regulator of angiogenesis in the CNS [30]. In the adult CNS, Nogo-A has also been shown to be a vital inhibitory factor of axonal regeneration and plasticity [30,31,32,33]. Nogo-B (55 kDa) is a shorter isoform than Nogo-A and has been shown to be expressed in cardiac myocytes and vascular cells in multiple cell types both in vitro and in vivo [34]. Expression of Nogo-B is reduced after injury in the femoral arteries of mice [25,34]. In addition, Nogo-B regulates the migration of endothelial cells in peripheral blood vessels, which results in vascular remodeling [35,36]. Nogo-C (25 kDa) is the smallest protein in the Nogo family [27,37] and is expressed in a variety of tissues and cells including neurons, liver cells, muscle cells, and cardiac cells [37]. Previous studies have shown that Nogo-C regulates apoptosis in cardiomyocytes during mouse myocardial infarction (MI) and that Nogo-C deficiency improves cardiac activity after MI [27]. In addition, Nogo-C expression is negatively correlated with tumor size and prognosis in hepatic carcinoma [37,38]. However, the endogenous role of Nogo-A in non-neural cells and the function of Nogo-A in inflammation are unknown.

It has been demonstrated that patients with Amyotrophic lateral sclerosis (ALS) and an experimental model of ALS showed up-regulation of Nogo-A in skeletal muscle [39]. Furthermore, the expression level of muscle Nogo-A in ALS patients is higher in type I fibers and is associated with the severity of nerve damage [40]. Based on these findings we hypothesized that Nogo-A might be involved in muscle injury, muscle degeneration and or in inflammatory cascade by mediating ER stress proteins.

In this study, we selected transcription factor CHOP as ER stress marker because it is the final downstream protein in ER stress signaling pathway. Furthermore, we investigated pro-inflammatory mediators and cytokines that are responsible for inflammatory disorders. We discussed the possible mechanism by which Nogo-A affects ER stress protein such as CHOP and inflammation in skeletal muscle and macrophages for the first time. To elucidate the role of Nogo-A in inflammatory mechanism, we utilized different in-vitro and in-vivo models including C2C12 cells, bone marrow-derived macrophages (BMDM) primary cells, Nogo-deficient mice, mdx mice and biopsies from myopathy and DMD patients. Thus, this study reveals the potential role of Nogo-A in the regulation of inflammatory mechanisms.

## 2. Materials and Methods

### 2.1. C2C12 Cell Culture

The murine myoblast cell line (C2C12) was cultured in Dulbecco’s Modified Eagle’s Medium (DMEM; Gibco-BRL, Grand Island, NY, USA) supplemented with 10% fetal bovine serum (FBS; Hyclone, Logan, UT, USA) and 1% penicillin/streptomycin (P/S). Cells were cultured in a humidified incubator containing 5% CO_2_ at 37 °C. C2C12 cells were grown until they were 60–70% confluent. Cells were then sub-cultured and grown for another 48 h. Finally, the cells were differentiated in 2% horse serum for 3 days, as previously described [41,42].

### 2.2. Recombinant Adenovirus and Si-RNA Transfection of C2C12 Cells

Adenovirus (Ad)-Nogo-A and Ad-CHOP were purchased from Vector Biolabs (Malvern, PA, USA). The small interfering (si) RNAs against Nogo-A and CHOP (si-cram, si-Nogo-A, and si-CHOP) were purchased from Bioneer Research (Seoul, Republic of Korea) and were transfected into cells using Lipofectamine 2000 reagent (Invitrogen, Carlsbad, CA, USA) according to the manufacturer’s instructions. The sequence of Nogo-A (si-RNA) was A CAA AGA GGA UUU AGU UUG UAG (forward) and UGC ACU ACA AAC UAA AUC CUC UUU G (reverse). For transfections, the cells were plated in 60 mm dishes at a density of 1 × 10^5^ cells in DMEM without antibiotics and allowed to grow for 24 h. When the cells became 40–50% confluent, the cells were transfected according to the manufacturer’s instructions.

### 2.3. Isolation, Culture, and Activation of Bone Marrow-Derived Macrophages

Macrophages were obtained from bone marrow with several modifications, as previously described [7,43]. Briefly, bone marrow cells were obtained by flushing the femur and tibia of 8-week-old C57BL/6 (wild type (WT)) and Nogo-knockout (KO) mice (*n* = 6). The femur and tibia were washed with 70% ethanol and then with PBS. Sterile scissors were used to cut both the knee and hip joints. The ends of the femur and tibia bones were also cut to obtain macrophages from the bone marrow. The bone marrow was flushed out in a 50 mL Falcon tube using a 26-gauge syringe and sterile PBS. The sample was then centrifuged at 3000× *g* for 5 min at 4 °C after which the cells were suspended in RPMI 1640 medium containing 15% conditioned medium from the L929 cell line as a source of macrophage colony stimulating factor (M-CSF). Cells were incubated for seven days and treated with lipopolysaccharide (LPS), an M1 inducer (100 ng/mL), or IL-4, an M2 inducer (20 ng/mL) for 24 h.

### 2.4. Animal Models Used in This Study

The experimental mice were housed in a pathogen-free facility at 21 ± 2°C with a humidity of 60 ± 10% under a 12 h light/dark cycle and feed and water were supplied ad libitum. For notexin experiment model, male 8–10-week-old WT (C57BL/6J) and global Nogo-KO (Nogo^−/−^) mice were used. The C57BL/6J mice were purchased from SLC incorporation (Hamamatsu, Japan) and Nogo isoforms-deficient (Nogo^−/−^) mice were kindly provided by Binhai Zheng (University of California, San Diego, CA, USA). For ER stress model, 10-week-old, male WT and Nogo-KO mice were used. Twelve-week-old male mdx mice (C57BL/10ScSn-Dmdmdx/J) were used for the DMD animal model. The mdx mice were generously gifted by Jacques P. Tremblay (CHUQ research center, Quebec City, Canada). All animal experiments and protocols were conducted with Institutional Animal Care and Use Committee (IACUC) guidelines and were approved by the Animal Care Committee of the Kyungpook National University, Daegu City 41566, Republic of Korea.

### 2.5. Muscle Injury

Muscle injury was induced by a single intramuscular (IM) injection of 20 μL of the myotoxic agent notexin (12.5 μg/mL, Latoxan, Valence, France), diluted in PBS, (or with 20 μL PBS as a control) into the gastrocnemius muscle of experimental mice. Briefly, WT and Nogo-KO mice (3 mice per group) were anesthetized after which both hind limbs were shaved. Notexin was injected into the right leg muscle, while the muscle of the left leg served as a control and was injected with PBS. Three days after notexin injection, the mice were euthanized and the gastrocnemius muscle was surgically isolated, as previously described [12,44]. The gastrocnemius muscle was cut in half as a cross-section, fixed in 4% paraformaldehyde (PFA) overnight, and subsequently transferred to 30% sucrose in PBS for 24 h. Using optimum cutting temperature (OCT) medium, the samples were embedded in a cryo block for histological analysis. The remaining half of the muscle sample was immediately frozen in liquid nitrogen for molecular analysis. The sample was subsequently stored at −80 °C until further analysis.

### 2.6. Induction of Endoplasmic Reticulum (ER) Stress Using Tunicamycin

WT and Nogo-KO mice were divided into the following 4 groups: WT mice without tunicamycin treatment (*n* = 3), WT mice treated with tunicamycin (*n* = 4), Nogo-KO mice without tunicamycin treatment (*n* = 4), and Nogo-KO mice treated with tunicamycin (*n* = 5). Tunicamycin was administered at a single dose of 1 µg/kg via intraperitoneal (IP) injection. Muscles were harvested 24 h after injection. Tunicamycin was prepared in DMSO and diluted in PBS to reduce the toxicity of DMSO in mice.

### 2.7. Human Myopathy

Muscle samples were harvested after diagnosis, and informed consent was obtained from all patients for the scientific use of their muscle biopsy specimens. The samples from patients with myopathy (male, *n* = 4 and female, *n* = 3) and those with DMD (male, *n* = 3) were collected according to the patient’s age (1.83 years, 2 years, 5 years, 5.1 years, 5.6 years, 15 years, 20 years, 46 years, 57 years, and 81 years). Five muscle biopsies were obtained for each group from age-matched healthy control subjects (*n* = 5), (1.5 years, 18 years, 26 years, 41 years, and 42 years). qRT-PCR and immunoblot analyses were performed on all muscle biopsy samples.

### 2.8. Quantitative Real-Time Polymerase Chain Reaction (qRT-PCR) Analysis

RNA was extracted from the gastrocnemius muscles (WT mice, Nogo-KO mice, mdx mice and DMD patients) and WT and Nogo-KO BMDM using TRIzol (Invitrogen, Carlsbad, CA, USA) according to the manufacturer’s instructions. Gene expression was measured by quantitative real-time polymerase chain reaction (qRT-PCR) using SYBR Green with low ROX (Enzynomics, catalog no. RT500S). Relative quantification of the target gene was determined by normalizing expression to that of the housekeeping gene GAPDH, which served as a control. The primer sequences used in this study are listed in Table 1. qRT-PCR data were analyzed using a CFX Connect Real-Time System (Bio-Rad).

### 2.9. Western Blot Analysis

Proteins were isolated and analyzed by immunoblotting. Briefly, the protein concentration in the samples was measured, samples were prepared in SDS and sample loading buffer, and heated for 10 min at 95 °C. Proteins were separated using 10% SDS-PAGE and immunoblotted onto membranes. The membranes were blocked with 1% bovine serum albumin (BSA) for 1 h and incubated with primary antibodies, including those against Nogo-A (Abcam, catalog no. ab62024), CHOP (Santa Cruz, catalog no. sc-71136), β-actin (Cell Signaling Technology, catalog no. 8457s), and GAPDH (Cell Signaling Technology, catalog no. 2118), overnight at 4°C. After a 1 h incubation with HRP-labeled secondary antibodies (Anti-rabbit-HRP, Cell Signaling Technology, catalog no. 7074s and Anti-mouse-HRP, Cell signaling Technology, catalog no. 7076s), the proteins were detected using enhanced chemiluminescence (ECL, Super Signal West Dura Extended Duration Substrate, catalog no. 34076) in an Amersham Imager 680 (GE Healthcare, Life Sciences). Blots were quantified using ImageJ software.

### 2.10. Immunofluorescence (IF) Assay

The immunofluorescence assay was performed with modifications, as previously described [45]. Briefly, cryosections and BMDM were washed with tris-buffered saline (TBS) and fixed in 4% paraformaldehyde (PFA) for 10 min. After washing, the samples were permeabilized with TBST (0.2% Triton X-100 in TBS) for 10 min and washed three times with TBS for 5 min. Samples were blocked using 2% BSA and incubated at 4°C overnight with primary antibodies, including rabbit anti-Nogo-A (Abcam, catalog no. ab62024), mouse anti-CD68 (Santa Cruz Biotechnology, catalog no. ab955), mouse anti-iNOS (Santa Cruz Biotechnology, ab49999), mouse anti-CD206 (Santa Cruz Biotechnology, catalog no. sc-58986), mouse anti-CHOP (Santa Cruz Biotechnology, sc-71136), and mouse anti-calnexin (Novus Biologicals, catalog no. NB300518). After three washes with TBS for 5 min, samples were incubated with secondary antibodies (donkey anti-mouse immunoglobulins (Alexa Fluor 488, Abcam, catalog no. ab150105) and donkey anti-rabbit immunoglobulins (Alexa Fluor 555, Abcam, catalog no. ab150066) for 1 h in the dark. The samples were mounted using ProLong™ Gold Antifade reagent containing DAPI to visualize the nuclei (Cell Signaling Technology, catalog no. 8961s) and were analyzed by confocal microscopy (ZEISS).

### 2.11. Histological Analysis

Gastrocnemius muscles from mice samples were rapidly fixed with 5% sucrose in 4% paraformaldehyde (PFA) for 24 h and subsequently transferred into 30% sucrose in PBS for 24 h. Samples were embedded in OCT compound for cryopreservation. Cryosections of 5 µm-thick tissues were cut for histological analysis. Sections of muscle were stained with hematoxylin and eosin (H&E). Stained tissue sections were visualized using a light microscope.

### 2.12. Measurement of Cytokine Levels

Control and notexin-treated WT and Nogo-KO gastrocnemius muscles were isolated, homogenized and supernatants were collected after centrifugation. IL-6 and TNF-α concentrations were measured using Mouse IL-6 and TNF-α ELISA kit (Life Technologies Corporation, Frederick, MD USA) according to the manufacturer’s protocol.

### 2.13. Flow Cytometry Analysis

BMDM were incubated for seven days and stimulated with lipopolysaccharide (LPS) (100 ng/mL) or IL-4 (20 ng/mL) for 24 h. BMDM were collected and washed twice in PBS and centrifuged at 1500× *g* for 3 min. Cells were incubated at 37 °C with primary antibodies against iNOS (Abcam, catalog no. ab49999) and CD206 (santa cruz, catalog no. sc-58986) for 1 h and were then washed with PBS. Finally, the cells were incubated with fluorochrome-labeled secondary antibodies in PBS for 30 min. After three washes in PBS, the cells were analyzed by flow cytometry.

### 2.14. Migration Assay

A migration assay was performed with modifications, as previously described [46]. Transwell chambers (6.5 mm diameter and 8 μm pore size) were obtained from Corning (catalog no. 3422). BMDM were harvested and suspended in RPMI supplemented with 10% FBS at a concentration of 2 × 10^4^ cells/well. Cells were seeded in serum-free medium into the upper chamber of a 24-well plate. The lower chambers were filled with RPMI medium containing 10% FBS. Cells were incubated overnight. Cells that had migrated to the reverse side of the Transwell membrane were fixed in 4% PFA and permeabilized with absolute methanol. Cells were stained with H&E, and non-migrated cells were removed using cotton swabs at which point the cells that had migrated were counted using a light microscope.

### 2.15. Phagocytosis Assay

BMDM were stimulated with Alexa Fluor 488-labeled zymosan fluorescent bioparticles (catalog no. z-23373). For flow cytometry, the BMDM were washed twice in PBS. Adherent cells were detached as a result of incubation with trypsin-EDTA for 5 min in the incubator and were subsequently centrifuged at 1500× *g* for 3 min. Cells were placed in the incubator and given 30 min to internalize the zymosan particles. Noninternalized particles were removed by three washes in cold PBS. The harvested cells were then washed and fixed in 4% paraformaldehyde. Cells were washed twice with PBS, placed in a FACS tube, and were immediately examined by flow cytometry.

### 2.16. Statistical Analysis

Statistical analysis was performed using GraphPad Prism 6.01 (GraphPad Software) program. Statistical significance was determined using Student’s *t*-test. Data are expressed as means and standard error of the mean (SEM). The statistical significance of data is denoted on the graphs by asterisks (*), with *p* values of * *p* < 0.05, ** *p* < 0.01, and *** *p* < 0.001.

## 3. Results

### 3.1. Nogo-A Regulates CHOP-Mediated Pro-Inflammatory Factor Expression in C2C12 Cells

**Figure 1:**
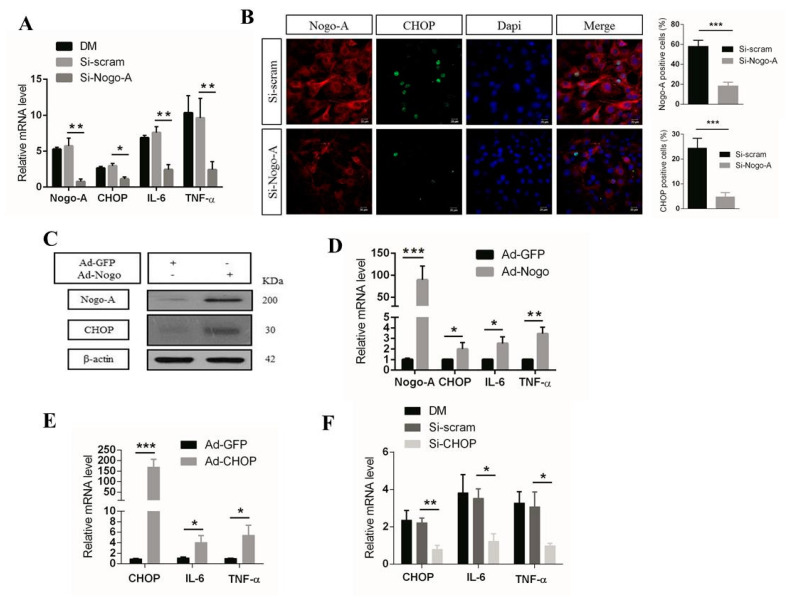
Nogo-A enhances CHOP expression and production of pro-inflammatory factors in C2C12 cells. (**A**) C2C12 cells were transfected with small interfering (si)-Nogo-A or si-scramble, after which the cells were differentiated (DM) for 3 days and then harvested for qPCR analysis of Nogo-A, CHOP, IL-6, and TNF-α expression levels. (**B**) Nogo-A and CHOP antibodies were used to stain transfected C2C12 cells for immunofluorescence (IF) analysis. (**C**) Cell extracts were analyzed by immunoblot (IB) analysis for Nogo-A, CHOP, and β-actin expression. (**D**) C2C12 cells were infected with adenovirus plasmid encoding green fluorescent protein (Ad-GFP) and Ad-Nogo-A (60 multiplicity of infection (MOI)) for 24 h. Nogo-A, CHOP, IL-6, and TNF-α mRNA levels were analyzed by qPCR. (**E**) Ad-GFP and Ad-CHOP (60 MOI) were used to infect C2C12 cells for 24 h. qPCR analysis of CHOP, IL-6, and TNF-α expression. (**F**) C2C12 cells were transfected with si-CHOP or si-control. After transfection for 36 h, the cells were differentiated for 3 days and then qPCR was performed to determine CHOP, IL-6, and TNF-α expression. In all, 40 μg of protein was used for IB. Data are shown as the mean ± standard error of the mean. Statistical significance was determined using Student’s *t*-test. The β-actin level was used for normalization of the expression levels. Data are denoted by asterisks where * *p* < 0.05, ** *p* < 0.01, *** *p* < 0.001. Alexa Fluor (AF)-488 and AF-555 were used as secondary antibodies. Scale bar, 10 µm, ×400 magnification.

First, we investigated whether Nogo-A affects the expression of pro-inflammatory factors in vitro. We evaluated the effects of Nogo-A using oligonucleotide small interfering RNA (si-Nogo-A) in C2C12 myoblast cells. We assessed differentiation-induced pro-inflammatory gene expression and found that Nogo-A knockdown led to significantly reduced levels of CHOP, IL-6, and TNF-α (Figure 1A), as JB Mdzomba et al. showed that Nogo-A antibodies inhibit inflammation [47].

Immunofluorescence (IF) data also showed that Nogo-A silencing remarkably decreased Nogo-A and CHOP expression (Figure 1B). These results indicate that Nogo-A could regulate CHOP, IL-6, and TNF-α expression in C2C12 cells.

To further assess the potential role of Nogo-A, we used an adenoviral delivery system to upregulate Nogo-A (Ad-Nogo), after which we assessed the expression levels of Nogo-A, CHOP, IL-6, and TNF-α. The protein levels of Nogo-A and CHOP were increased in the Ad-Nogo-infected group relative to the control group (Figure 1C). The mRNA levels of Nogo-A, CHOP, IL-6, and TNF-α were also significantly increased after infection with Ad-Nogo relative to the control (Ad-GFP) (Figure 1D). These data suggest that Nogo-A enhances activation of the CHOP signaling pathway, which could induce pro-inflammatory gene transcription.

To examine the role of CHOP in the regulation of pro-inflammatory factor expression, we assessed CHOP overexpression using an adenoviral delivery system (Ad-CHOP) and CHOP knockdown using small interfering RNA (si-CHOP) in C2C12 cells. We demonstrated that high CHOP expression by Ad-CHOP significantly promoted IL-6 and TNF-α mRNA expression compared with the Ad-GFP (control) (Figure 1E).

In contrast, the expression of pro-inflammatory factors was dramatically reduced by CHOP silencing (si-CHOP) (Figure 1F), as previously described [23,48]. Moreover, LX Jia et al. reported that the mRNA levels of IL-6, IL-1β, and CCL2 were significantly decreased in CHOP knockout mice [49]. Our results suggest that Nogo-A regulates CHOP-mediated expression of inflammatory factors.

### 3.2. Nogo Deficiency Suppresses Expression of Pro-Inflammatory Factors in BMDM

**Figure 2:**
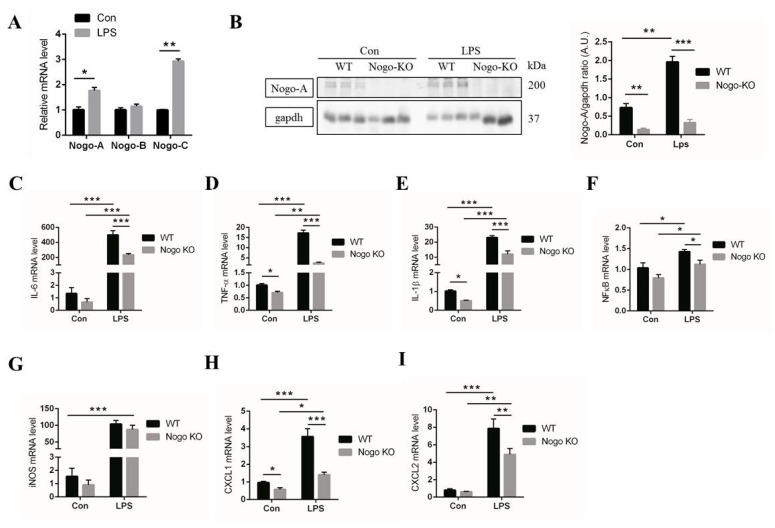
Nogo deficiency inhibits pro-inflammatory gene expression in BMDM after lipopolysaccharide (LPS) treatment. (**A**) Nogo-A and C, but not Nogo-B, are upregulated in LPS-treated bone marrow-derived macrophages (BMDM) compared with the levels in control BMDM (*n* = 3). (**B**) IB data of control and LPS-treated BMDM. Nogo-A was increased in response to LPS treatment in WT BMDM compared with the levels in Nogo-KO BMDM (*n* = 3). (**C**–**I**) mRNA levels of pro-inflammatory factors including IL-6, TNF-α, IL-1β, nuclear factor kappa B (NF-κB), iNOS, CXCL1, and CXCL2 in control and LPS-treated WT and Nogo-KO BMDM (*n* = 4). In all, 30 μg of protein was used in IB. GAPDH was used for normalization. Data are shown as the mean ± standard error of the mean. Statistical significance was determined using Student’s *t*-test. Data are denoted by asterisks, where * *p* < 0.05, ** *p* < 0.01, and *** *p* < 0.001.

We investigated the critical role of Nogo-A in inflammation in bone marrow-derived macrophages (BMDM). We isolated BMDM from WT and Nogo-KO mice and cultured them for seven days (Figure S1A). IF staining for the macrophage marker CD68 indicated that the isolated cells are macrophages (Figure S1B). Using qPCR, we found that Nogo-A and Nogo-C, but not Nogo-B, were significantly activated in BMDM that were treated with LPS (Figure 2A). Using Western blot, we observed that Nogo-A levels were significantly elevated in LPS-stimulated BMDM compared with control (unstimulated) BMDM (Figure 2B).

We next determined whether Nogo-A regulates the expression of pro-inflammatory factors in BMDM from WT and Nogo-KO mice. We found that LPS-treated Nogo-KO BMDM expressed significantly decreased mRNA levels of IL-6, TNF-α, IL-1β, NF-κB, CXCL1, and CXCL2 compared with WT BMDM, whereas iNOS was not significantly downregulated in LPS-treated Nogo-KO BMDM compared with WT BMDM (Figure 2C–I), as previously described [50]. These results suggest that Nogo-A may be involved in the activation of pro-inflammatory factors in LPS-treated WT BMDM. In contrast, the expression levels of anti-inflammatory (M2) factors, including arginase-1, CD206, and IL-10, were not significantly elevated after LPS treatment (Figure S2A–C).

We next determined the role of Nogo-A in BMDM activation using the strong M2 inducer IL-4. IF staining showed that Nogo-A was expressed in control WT BMDM but that CD206 was not expressed in control BMDM derived from WT and Nogo-KO mice (Figure S2D). In the IL-4-treated group, CD206 expression was slightly upregulated in Nogo-KO BMDM compared with WT BMDM (Figure S2E). In addition, flow cytometry showed that, after IL-4 treatment, CD206 expression was similarly elevated in Nogo-KO BMDM compared with WT BMDM, but this difference was not significant (Figure S2F).

### 3.3. Nogo Deficiency Suppresses CHOP Signaling and Migration of BMDM

**Figure 3:**
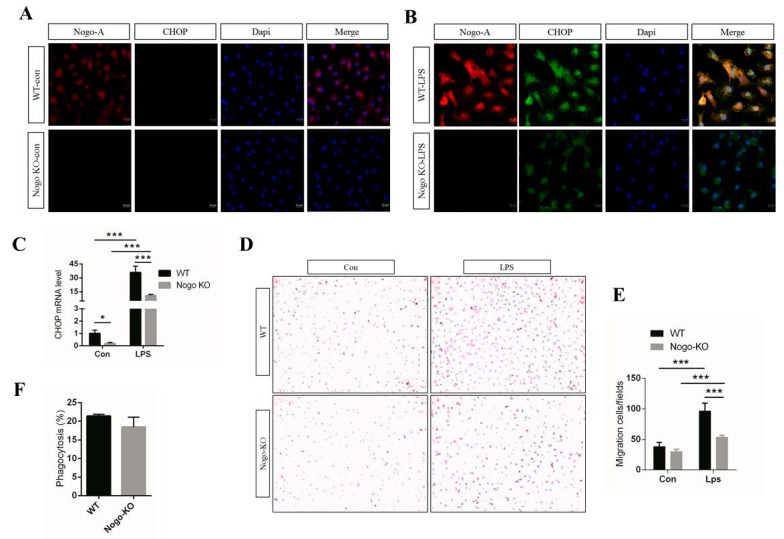
Nogo deficiency inhibits CHOP signaling and migration of BMDM after LPS treatment. (**A**) IF results of Nogo-A and CHOP in control WT BMDM and Nogo-KO BMDM (*n* = 3). Nogo-A is expressed in control WT BMDM, while CHOP expression is absent in control WT and Nogo-KO BMDM. (**B**) IF results show that Nogo-A (red) and CHOP (green) are increased in LPS-treated WT BMDM compared with the levels in Nogo-KO BMDM. (**C**) Level of CHOP mRNA is upregulated in LPS-treated WT BMDM compared with LPS-treated Nogo-KO BMDM (*n* = 4). (**D**) Migration assay using WT and Nogo-KO BMDM (*n* = 3). Nogo-KO BMDM exhibit a lower migration ability compared with WT BMDM after LPS treatment (100 ng/mL) for 24 h. (**E**) Quantification of BMDM migration reveals a significantly lower migration ability in LPS-treated Nogo-KO MMDM compared with WT BMDM (*n* = 3). No significant difference was observed in migration activity between control WT and Nogo-KO BMDM. (**F)** Phagocytosis by macrophages from WT and Nogo-KO BMDM after treatment with fluorescent bioparticles of the pro-inflammatory cytokine inducer zymosan (*n* = 3). The numbers of phagocytes were analyzed by flow cytometry. Data are shown as the mean ± standard error of the mean. Statistical significance was determined using Student’s *t*-test. Data are denoted by asterisks, where * *p* < 0.05 and *** *p* < 0.001. Alexa Fluor (AF)-555 and AF-488 were used as secondary antibodies. Scale bar, 10 µm, 400× magnification.

To develop a better understanding of the molecular relationship between Nogo-A and CHOP, we performed IF staining and found that Nogo-A was expressed in control WT BMDM and that CHOP was not expressed in either control WT or Nogo-KO BMDM (Figure 3A). However, when Nogo-KO BMDM were treated with LPS, CHOP expression was downregulated relative to that in WT BMDM (Figure 3B).

In addition, we used qPCR to measure the levels of CHOP in control BMDM and in LPS-treated BMDM. In LPS-treated Nogo-KO BMDM, CHOP expression was significantly reduced (Figure 3C). Moreover, IF results demonstrated that Nogo-A co-localized with calnexin, an ER marker (Figure S3A,B). These results suggest that CHOP expression is reduced in Nogo-KO BMDM.

We next assessed whether Nogo-A affects the inflammatory response through the inflammation inducer LPS, as determined by the migration activity of macrophages. Interestingly, the migration assay indicated that the migration activity of BMDM was significantly decreased in LPS-treated Nogo-KO BMDM relative to WT BMDM (Figure 3D,E). In contrast, BMDM migration did not significantly differ between the control WT and the Nogo-KO BMDM.

Phagocytic activity is a fundamental biological mechanism of macrophages. Phagocytosis with zymosan is a popular technique used by macrophages [51]. To assess the involvement of the phagocytic activity of macrophages, we used an inducer of pro-inflammatory factors (zymosan) along with Alexa Fluor 488-labeled fluorescent bioparticles. Using FACS, we found that phagocytic activity was higher in WT BMDM compared with Nogo-KO BMDM, although the difference was not statistically significant (Figure 3F). These data suggest that Nogo deficiency prevents the migration of BMDM derived from LPS-stimulated Nogo-KO mice.

### 3.4. Nogo-A, CHOP, and Pro-Inflammatory Factors Are Upregulated in Injured Muscle

**Figure 4:**
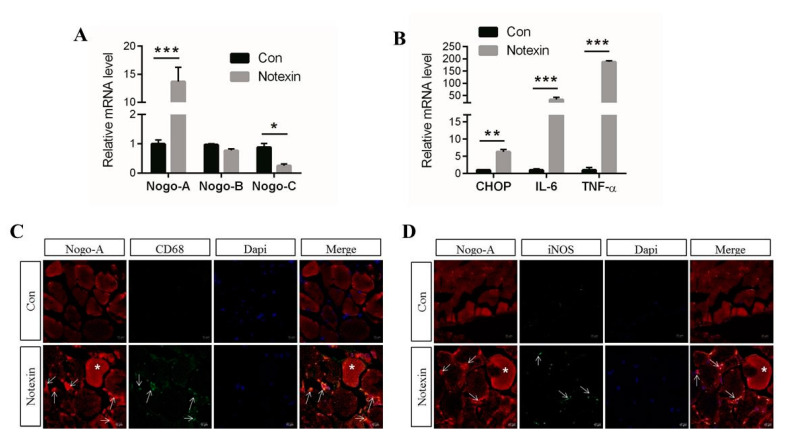
Expression of Nogo-A, CHOP, and pro-inflammatory factors is increased in muscle from notexin-treated mice. (**A**) Nogo-A is upregulated in injured muscle, while levels of Nogo-B and C are not significantly altered. Notexin-injured muscle expresses higher levels of Nogo-A compared with those in the WT control (*n* = 3). (**B**) Expression of Nogo-A, CHOP, IL-6, and TNF-α is upregulated in notexin-injured muscle (*n* = 3). (**C**) Notexin-injured muscle was immunostained using antibodies against Nogo-A and CD68 (*n* = 3). Nogo-A is localized to muscle fibers (asterisk) and colocalizes with CD68 (arrow). (**D**) Notexin-injured muscle was immunopositive for Nogo-A and iNOS (*n* = 3). Nogo-A is localized to muscle fibers (asterisk) and colocalizes with iNOS (arrow) 3 days after a single intramuscular injection of notexin (12.5 μg/mL, 20 μL). Data were normalized to RNA expression of GAPDH. Data are shown as the mean ± standard error of the mean. The statistical significance was determined using Student’s *t*-test. Data are denoted by asterisks, where * *p* < 0.05, ** *p* < 0.01, and *** *p* < 0.001. Secondary antibodies used were Alexa Fluor (AF)-555 and AF-488. Scale bar, 10 µm, ×400 magnification.

Next, we aimed to determine the role of Nogo-A in muscle inflammation. To achieve this, we used notexin, which is a myotoxic agent used to induce muscle injury [52,53]. We tested whether Nogo-A was activated three days after notexin-induced injury in muscle. We found that Nogo-A levels were significantly increased in notexin-injured muscle, while Nogo-B was not significantly altered in either the control or the notexin-treated muscle; however, Nogo-C was reduced in notexin-treated muscle compared with the control (Figure 4A). Recent research has noted that retinal excitotoxicity results in the upregulation of Nogo-A expression [47]. Our results suggest that only Nogo-A, but not Nogo-B or Nogo-C, is activated in injured muscle.

We also found that levels of CHOP and pro-inflammatory cytokines, such as IL-6 and TNF-α, were also elevated in the notexin-treated mice (Figure 4B). These results support the mRNA expression of IL-6 and TNF-α. A previous study showed that CHOP contributes to cytokine-induced pro-inflammatory responses [54].

Using immunofluorescence (IF) analysis, we found that levels of Nogo-A, cluster of differentiation (CD)-68 (a marker of macrophages), and inducible nitric oxide synthase (iNOS) (a pro-inflammatory marker), were increased in notexin-injured muscle (Figure 4C,D). In addition, the mRNA levels of pro-inflammatory mediators and cytokines including iNOS, IL-1β, and NF-κB and chemokines including CXCL1 and CXCL2 were also upregulated in notexin-treated mice compared with untreated mice (Figure S4). Together, these results suggest that the levels of Nogo-A, CHOP, pro-inflammatory cytokines, and chemokines are increased in notexin-induced muscle injury.

### 3.5. Pro-Inflammatory Factor Expression Mediated by CHOP Signaling IS Nogo-A-Dependent

**Figure 5:**
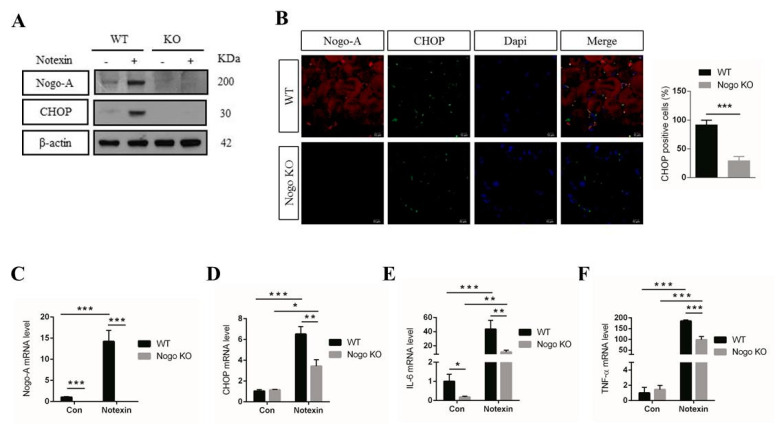
Pro-inflammatory factors mediated by CHOP signaling. (**A**) Nogo-A, CHOP, and β-actin protein expression levels in muscle from notexin-treated WT mice compared with muscle from notexin-treated Nogo-KO mice (*n* = 3). (**B**) Expression of CHOP in injured muscle from WT mice compared with its expression in injured muscle from Nogo-KO mice (*n* = 3) by immunofluorescence (IF). (**C**–**F**) Nogo-A, CHOP, IL-6, and TNF-α mRNA expression levels in gastrocnemius muscle isolated from WT (*n* = 3) and Nogo-KO mice (*n* = 3) 3 days after a single intramuscular injection of notexin (12.5 μg/mL). Data were normalized to RNA expression of GAPDH. In all, 40 μg of protein was used for immunoblot (IB) experiments. Data are shown as the mean ± standard error of the mean. Statistical significance was determined using Student’s *t*-test. Secondary antibodies used were Alexa Fluor (AF)-555 and AF-488. Significant data are denoted by asterisks where * *p* < 0.05, ** *p* < 0.01, and *** *p* < 0.001. Scale bar, 10 µm, ×400 magnification.

We next aimed to determine the role of Nogo-A in the regulation of the inflammatory process. To this end, we used wild type (WT) and Nogo-knockout (KO) mice and measured the levels of Nogo-A and CHOP in the muscle of mice treated with notexin. An immunoblot (IB) analysis revealed significant upregulation of Nogo-A and CHOP in WT notexin-treated muscle relative to Nogo-KO mice (Figure 5A).

Using IF, we observed higher levels of CHOP expression in WT muscle compared with Nogo-KO muscle after notexin treatment (Figure 5B). We also found significantly higher levels of Nogo-A mRNA in notexin-treated WT muscle compared with untreated control WT muscle (Figure 5C). CHOP, IL-6, and TNF-α mRNA levels were significantly elevated in notexin-treated WT muscle compared with notexin-treated Nogo-KO muscle, while IL-6 levels were increased in WT control mice compared with Nogo-KO control mice (Figure 5D–F). A previous study showed that Nogo-A antibody treatment decreased the expression of inflammation-related genes [47]. These results suggest that absence of Nogo-A reduces CHOP, IL-6, and TNF-α expression. We have done ELISA to confirm the IL-6 and TNF-α released (Figure S5). H&E staining showed that inflammatory cells infiltrated in notexin-treated WT and Nogo-KO mice compared with control groups (Figure S6).

**Figure 6:**
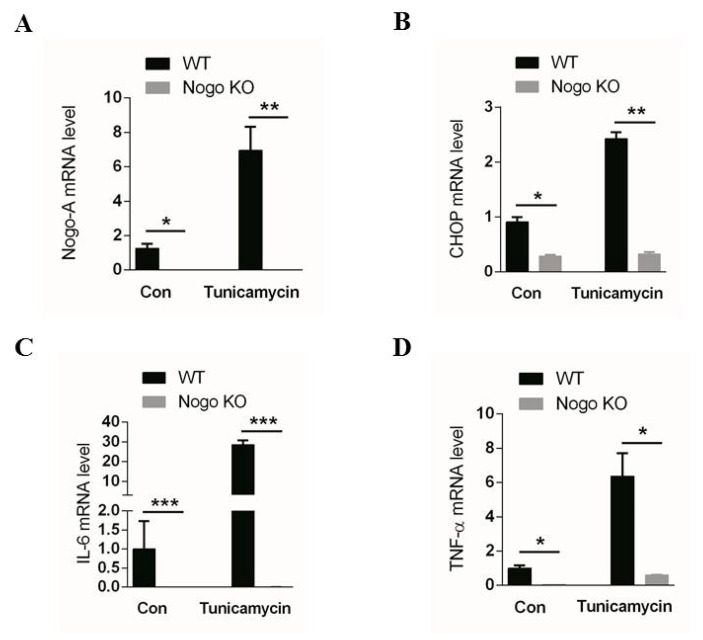
Tunicamycin-induced endoplasmic reticulum (ER) stress was mediated by the Nogo-A-CHOP pathway. (**A**) The Nogo-A expression level is upregulated in the skeletal muscle of tunicamycin-treated WT mice compared with Nogo-KO mice. (**B**) CHOP is highly expressed in tunicamycin-treated skeletal muscle of WT mice compared with Nogo-KO mice. (**C**,**D**) IL-6 and TNF-α are highly expressed in skeletal muscle of tunicamycin-treated WT mice compared with skeletal muscle of Nogo-KO mice. Data are shown as the mean ± standard error of the mean. Statistical significance was determined using Student’s *t*-test. Data are denoted by asterisks where * *p* < 0.05, ** *p* < 0.01, *** *p* < 0.001.

To further examine the role of Nogo-A in ER stress, we administered a single dose (1 µg/kg) of tunicamycin, a pharmacological ER stress inducer, via intraperitoneal (IP) injection into WT and Nogo-KO mice. Using qPCR, we found that the levels of Nogo-A, CHOP, IL-6, and TNF-α were significantly low in Nogo-KO mice compared to WT (Figure 6A–D).

### 3.6. Expression of Nogo-A, CHOP, and Pro-Inflammatory Factors Is Increased in Mdx Mice and Human DMD Patients

**Figure 7:**
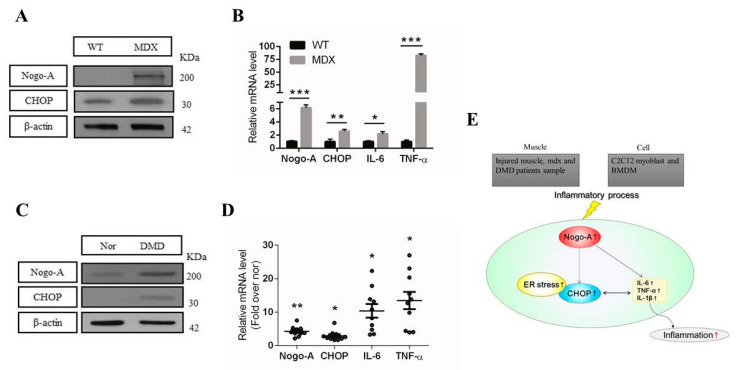
Nogo-A, CHOP, and pro-inflammatory factors are upregulated in mdx mice and Duchenne muscle dystrophy (DMD) patient samples. (**A**) IB analysis of Nogo-A, CHOP, and β-actin in the skeletal muscle of WT and mdx mice. (**B**) qPCR analysis of mRNA from the skeletal muscle of 12-week-old WT and mdx mice (*n* = 4). (**C**) Tissue extracts from DMD and myopathy patients and normal subjects were used in the IB analysis with Nogo-A, CHOP, and β-actin antibodies. (**D**) The qPCR analyses were performed on biopsy samples of DMD and myopathy patients and normal subjects. (**E**) Proposed model of the role of Nogo-A in the regulation of inflammation. Nogo-A is activated in muscle from notexin-treated mice, mdx mice, DMD patients and in LPS-treated BMDM. Subsequently, Nogo-A expression may be accompanied by CHOP activation and can also activate pro-inflammatory cytokines and chemokines in injured or degenerated muscle and in LPS-stimulated BMDM. We conclude that Nogo-A exerts inflammatory effects. In all, 40 μg of protein was used for the IB experiments. The statistical significance was determined using Student’s *t*-test. Error bars represent the standard error of the mean. Data are denoted by asterisks where * *p* < 0.05, ** *p* < 0.01, and *** *p* < 0.001.

We assessed the activation of Nogo-A and CHOP in a DMD mouse model (mdx mice). Western blot showed that Nogo-A and CHOP protein levels were dramatically upregulated in mdx mice (Figure 7A). In mdx mice, we found that the mRNA levels of Nogo-A, CHOP, IL-6, and TNF-α were also significantly increased compared with those in WT mice (Figure 7B), as previously reported [55,56]. Inflammatory mediators have also been shown to participate in fibrosis in mdx mice [57]. These data suggest that Nogo-A, CHOP, IL-6, and TNF-α expression is increased in mdx mice compared with WT mice.

To verify the clinical relevance of higher expression of Nogo-A, CHOP, and pro-inflammatory genes in DMD patients, we performed an immunoblot analysis and qPCR. Using Western blot, we observed significantly increased levels of Nogo-A and CHOP in DMD patient samples compared with healthy donors (Figure 7C). Finally, we found that myopathy and DMD patients group (n=10) had significantly elevated levels of Nogo-A, CHOP, IL-6, and TNF-α mRNA compared with healthy subjects group (n=5) (Figure 7D). Taken together, these data suggest that Nogo-A promotes inflammation in both mdx mice, myopathy and DMD patients.

## 4. Discussion

Here, we discussed our understanding of the inflammatory mechanisms of Nogo-A in different models. Based on our results, we summarized the regulation of Nogo-A in inflammation in Figure 7E. An earlier study has shown that Nogo-A was upregulated in skeletal muscles of patients with Amyotrophic lateral sclerosis (ALS) and experimental models of ALS [39]. Moreover, it has also been stated that Nogo-A was remarkably elevated in muscles of ALS patients in type I fibers which are associated with the severity of nerve damage [40]. In addition, previous results showed that the ER stress proteins including CHOP were upregulated in gastrocnemius muscle of SOD1 murine model of ALS [58]. CHOP is ER stress marker protein that acts as a transcription factor resulting in regulation of pro-inflammatory cytokines [14,23]. Relying on these findings, we designed our experiments to examine the expression level of Nogo-A, CHOP and inflammatory cytokines in in vitro and in vivo models.

To examine the possible role of Nogo-A in ER stress and inflammation in murine myocytes (C2C12), we knocked down Nogo-A using si-RNA specific for Nogo-A. In addition, we overexpressed Nogo-A in C2C12 using adenovirus. Results showed that Nogo-A is highly expressed during 3 days of differentiation. Interestingly, knocking down of Nogo-A during 3 days of differentiation resulted in remarkable reduction in mRNA expression level of CHOP and pro-inflammatory cytokines such as IL-6, and TNF-α (Figure 1A). A previous study also stated that Nogo-A silencing led to the downregulation of IL-6 and TNF-α in LPS-stimulated PC12 cells [50] which is consistent with our result, however cell line is different. Moreover, immunofluorescence (IF) staining of Nogo-A knocked down in C2C12 showed a significant decrease in CHOP expression compared to si-scram (Figure 1B). On the contrary, adenovirus-based overexpression of Nogo-A (Ad-Nogo) in C2C12 cells led to the increased mRNA expression of CHOP, IL-6, and TNF-α and protein levels of Nogo-A and CHOP were elevated in the Ad-Nogo-infected group (Figure 1C,D). According to the aforementioned data, silencing of Nogo-A results in decreased expression of CHOP, IL-6 and TNF-α, while overexpression of Nogo-A results in increased expression of CHOP, IL-6 and TNF-α. These findings show the strong relationship among Nogo-A, CHOP and pro-inflammatory cytokines.

In order to verify that CHOP regulates downstream expression of inflammatory cytokines in C2C12, overexpression and silencing of CHOP was performed (Figure 1E,F). Overexpression was done using Ad-CHOP which led to an increase in mRNA expression levels of IL-6 and TNF-α, as previously reported [59]. In contrast, CHOP silencing using si-CHOP dramatically reduced the expression of IL-6 and TNF-α. Previous studies have shown that the inflammatory response was significantly reduced in CHOP knockout mice [23,48,49]. These results indicate that CHOP regulates the expression of pro-inflammatory cytokines in C2C12 cells.

Pro-inflammatory macrophages release greater levels of chemical substances including pro-inflammatory cytokines, enzymes, and chemokines such as IL-6, IL-1β, TNF-α, iNOS, CXCL1, and CXCL2 [8,60]. TNF-α is mainly induced by activation of M1 macrophages and can lead to the expression of other cytokines by M1 macrophages, including IL-6, and can regulate the inflammatory process [61,62]. Real time PCR and Western blot are good techniques for measuring the expression levels of Nogo-A, CHOP and pro-inflammatory cytokines in the whole injured skeletal muscle. However, these techniques cannot differentiate if the expression of Nogo-A, CHOP and pro-inflammatory cytokines are by muscle cells or inflammatory cells. Our IF staining showed that increased expression of Nogo-A and CHOP not only by myofibers but also by activated muscle macrophages (Figure 4C,D).

Muscle-resident macrophages can generate from multiple origins such as embryonic or adult hematopoiesis and they play vital roles in regulating biological processes including tissue remodeling, tissue homeostasis, tissue repair and immune responses [63]. Although the origins of bone marrow-derived macrophages (BMDM) and tissue-resident macrophages are not exactly the same, functions are similar in both types of macrophages. When BMDM are activated and stimulated by LPS they generate the pro-inflammatory cytokines that are also produced by tissue-resident macrophages. We therefore isolated macrophages from bone marrow of WT and Nogo-deficient mice before differentiating and stimulating them with lipopolysaccharide (LPS) to confirm that Nogo-A has the same function of regulating CHOP and pro-inflammatory cytokines expression in macrophages as in muscle cells.

Expression of Nogo-A, CHOP and pro-inflammatory cytokines were compared between WT and Nogo-deficient BMDM. Interestingly, WT BMDM responded to LPS stimulation by significantly high expression of Nogo-A, CHOP and pro-inflammatory mediators and cytokines compared to Nogo-KO BMDM. Moreover, iNOS and NF-κB pathways that contribute to cytokine induction were found to be upregulated in WT BMDM comparing to Nogo-KO BMDM (Figure 2). These findings confirmed that Nogo-A is essential for activation of inflammation in BMDM as well as injured muscle. Additionally, Nogo-C mRNA expression level was higher than Nogo-A in LPS-stimulated BMDM. This finding might suggest a possible synchronization between Nogo-A and C in stimulation of BMDM. However, Nogo-C was downregulated in notexin-injured muscles while Nogo-A was elevated (Figure 4A). Depending on this finding we can understand that Nogo-A regulates inflammation in injured muscles, particularly myofibers, while stimulation of inflammation in macrophages is regulated by Nogo-A as well as Nogo-C. Nogo-C elevation in macrophages could be due to apoptosis, as previously described [37].

In the current study, it is suggested that Nogo regulates chemotaxis in response to LPS. The mRNA expression for CXCL1 and CXCL2, major chemokines which are involved in chemotaxis and spreading of cell, are also significantly reduced in Nogo-KO BMDM (Figure 2H,I). In addition, previous research has shown that cytokine secretion facilitates macrophage migration [64]. Our data showed that migration activity of LPS-treated WT BMDM is significantly higher than Nogo-KO BMDM (Figure 3D,E). Therefore, Nogo-A may affect the migration of macrophages in LPS-stimulated BMDM. We hypothesized that an increased number of pro-inflammatory macrophages after stimulation of WT BMDM by LPS may be linked to increased phagocytosis. Moreover, our phagocytosis assay revealed a higher trend of phagocytic activity in WT BMDM compared with Nogo-KO BMDM (Figure 3F). Taken together, our data suggest that Nogo-A may regulate the migration and phagocytic activity of macrophages in LPS-stimulated BMDM.

In this study, investigation of notexin-injured muscles revealed increased expression of Nogo-A, CHOP, and pro-inflammatory factors in WT mice three days after notexin treatment (Figure 4). However, notexin-treated Nogo-KO muscles caused a reduction in CHOP expression and inflammatory cytokines compared to WT injured-muscle (Figure 5). Previous study showed that Nogo-A shapes and stabilizes ER tubules [26]. Thus, loss of Nogo-A might affect production of ER-related proteins including CHOP which results in reducing inflammation. Previous work has shown that cytokines induce ER stress in vitro, as observed by increased levels of CHOP [65]. Similarly, our results suggest that upregulation of IL-6 and TNF-α in injured muscle is associated with CHOP induction and may partially involve Nogo-A in the inflammatory process.

Mice were injected with tunicamycin which is a chemical that is used to induce ER stress [66]. Real time PCR showed upregulation of Nogo-A mRNA expression in tunicamycin-treated WT muscle comparing to non-treated WT muscle (Figure 6A). However, tunicamycin-treated Nogo-KO muscle showed low expression levels of CHOP, IL-6 and TNF-α comparing to tunicamycin-treated WT muscle (Figure 6B–D). These results show the significance of Nogo-A in induction of ER stress and that absence of Nogo-A ameliorates muscle inflammation due to ER stress.

In our recent study, we showed that Nogo-A was significantly elevated in mdx mice [67]. Investigation of the skeletal muscle in mdx mice showed increased mRNA of Nogo-A, CHOP, IL-6, and TNF-α compared with WT mice (Figure 7B). Moreover, Western blot result also showed increased protein expression of Nogo-A and CHOP in mdx mice compared with WT mice (Figure 7A). Our data suggest the critical role of Nogo-A in regulation of ER stress and inflammation in a muscular dystrophy model.

Muscle biopsies from myopathy and DMD patients had significant increases in Nogo-A, CHOP, IL-6, and TNF-α expression at both the mRNA and protein levels (Figure 7C,D). In mdx mice and DMD patients, elevated levels of pro-inflammatory cytokines were observed in the blood at pre-symptomatic stages of the disease [12,55,56,68]. Thus, the release of specific pro-inflammatory cytokines may stimulate the production of reactive oxygen species (ROS), which would enhance cellular damage in DMD [12]. Our findings suggest that Nogo-A contributes by regulating the inflammatory process for injured muscle which might affect muscle regeneration.

## 5. Conclusions

In summary, our results demonstrated that silencing of Nogo-A resulted in alleviation of CHOP and pro-inflammatory cytokines in C2C12. Overexpression of Nogo-A in C2C12 led to increased mRNA expression of CHOP, IL-6, and TNF-α. LPS-stimulated Nogo-deficient macrophages showed lower expression of CHOP and pro-inflammatory cytokines. Nogo-A was markedly upregulated in notexin-injured WT muscle compared to non-injured WT muscle. The expression levels of CHOP and pro-inflammatory cytokines were low in notexin-injured Nogo-KO muscle compared to injured WT muscle. Nogo-A was markedly upregulated in tunicamycin-injured WT muscle compared to non-injured WT muscle. The expression levels of CHOP and pro-inflammatory cytokines were low in tunicamycin-injured Nogo-KO muscle compared to injured WT muscle. Expression of Nogo-A, CHOP and pro-inflammatory cytokines were significantly high in mdx muscle and DMD patients muscles. Our findings indicate that Nogo-A might be a potential therapeutic target for the treatment of inflammatory diseases, such as myopathies, but more studies are needed to better understand the mechanisms of Nogo-A in inflammatory diseases.

## Figures and Tables

**Table 1:** Primer sequences used in qRT-PCR.

Gene Name	Forward Primer	Reverse Primer
Nogo-A	CTC AGT GGA TGA GAC CCT TTT TGC	CAG TGT TAC CTG GCT GCT CCT
Nogo-B	TC AGT GGT TGT TGA CCT CC	GC CGT TAC ACT GAC AAT GC
Nogo-C	GAT CGT GGC AAG AAA TGG ACG	AGC AGG AAT AAG CTG GCA CC
IL-6	GGA GAC TTC ACA GAG GAT AC	ATC TCT CTG AAG GAC TCT GG
TNF-α	TTC TCA TTC CTG CTT GTG GC	TTG AGA TCC ATG CCG TTG
IL-1β	GC ACT ACA GGC TCC GAG ATG AAC	TT GTC GTT GCT TGG TTC TCC TTG T
iNOS	TT CAC CCA GTT GTG CAT CGT CCT A	TC CAT GGT CAC CTC CAA CAC AAG A
NF-κB	GCC TAC CCG AAA CTC AAC TTC	CTC TTT GGA ACA GGT GCA GAC
Cxcl1	CC GAA GTC ATA GCC ACA CTC A	GT GCC ATC AGA GCA GTC TGT
Cxcl2	GAA GTC ATA GCC ACT CTC AAG G	CCT CCT TTC CAG GTC AGT TAG C
CHOP	CCT GAC GAC AGA GTG TTC CAG	CTC CTG CAG ATC CTC ATA CCA
CD206	CA GGT GTG GGC TCA GGT AGT	TG TGG TGA GCT GAA AGG TGA
Arginase-1	CT CCA AGC CAA AGT CCT TAG AG	AG GAG CTG TCA TTA GGG ACA TC
IL-10	GCC TTG CAG AAA AGA GAG CT	AAA GAA AGT CTT CAC CTG GC
Gapdh	TCA ATG AAG GGG TCG TTG AT	CGT CCC GTA GAC AAA ATG GT

## Data Availability

All data generated or analyzed during this study are included in this published article (and its supplementary information files).

## Supplementary Materials

The following are available online at https://www.mdpi.com/2073-4409/10/2/282/s1, Figure S1: Isolation of bone marrow-derived macrophages (BMDM) from WT and Nogo-KO mice, Figure S2: Expression of anti-inflammatory factors in BMDM, Figure S3: Nogo-A co-localized with calnexin, an endoplasmic reticulum (ER) marker in BMDM, Figure S4: Expression of pro-inflammatory markers in the gastrocnemius muscle from notexin-treated mice, Figure S5: Nogo regulates pro-inflammatory cytokines in notexin-induced muscle damage in mice, Figure S6: Histological features of control and notexin-treated muscle in WT and Nogo-KO mice.
