# Supplementary material for: Nogo-A Is Critical for Pro-Inflammatory Gene Regulation in Myocytes and Macrophages
Source: Cells. 2021 Jan 31;10(2):282. doi: 10.3390/cells10020282 (PMC7912613; doi:10.3390/cells10020282)
Supplement: Supplementary file 1 [file cells-10-00282-s001.pdf]

Supplementary Figure Legends

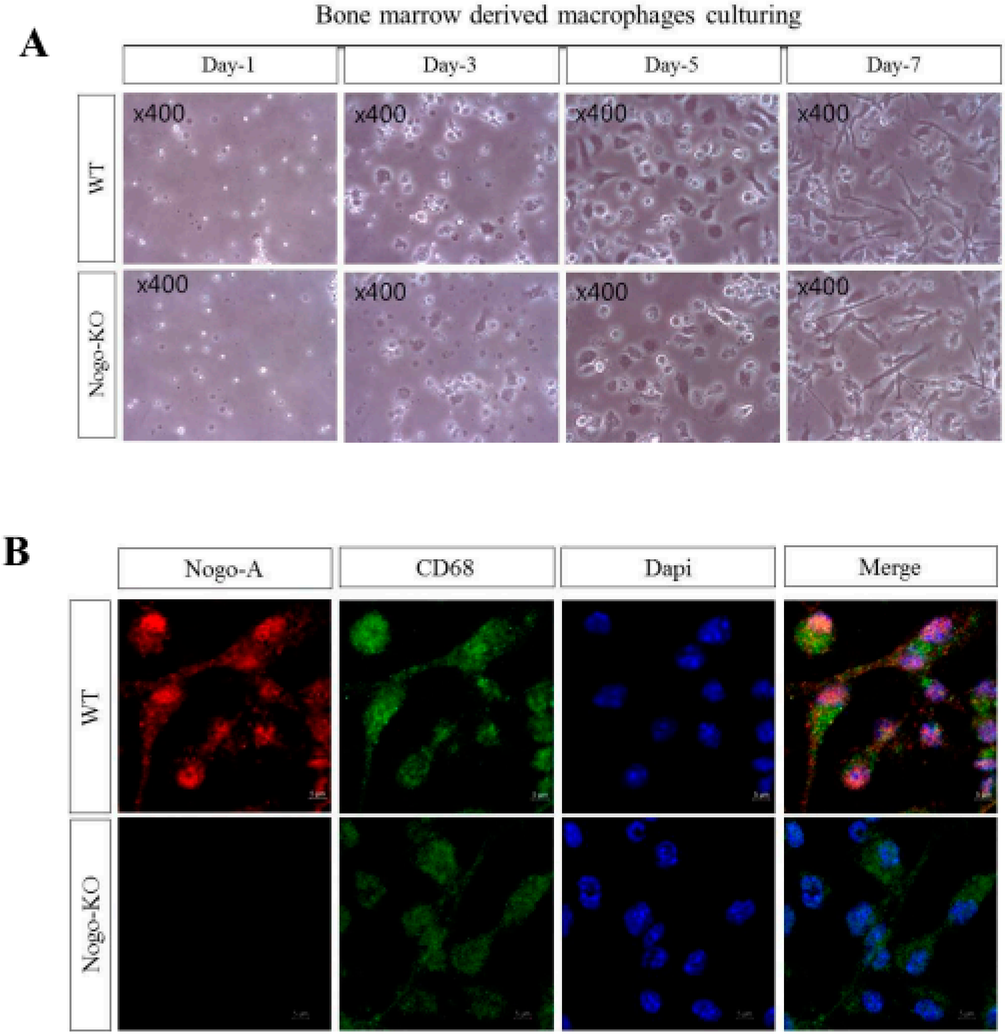

Figure S1: Isolation of bone marrow-derived macrophages (BMDM) from WT and Nogo-KO mice. (A) Macrophages obtained from WT and Nogo-KO mice were cultured for seven days (n = 3). After culture, BMDM were stimulated with pro-inflammatory mediators, lipopolysaccharide (LPS), and the anti-inflammatory mediator IL-4 for 24 h. (B) IF staining for the ER protein Nogo-A (red) and the macrophage marker CD68 (green). After 7 days of culture of LPS-treated (100 ng/mL) and IL-4-treated (20 ng/mL) BMDM, a molecular analysis was performed. Alexa Fluor (AF)-488 and AF-555 were used as secondary antibodies. DAPI was used for nuclear staining. Scale bar, 5 μm, ×1000 magnification.

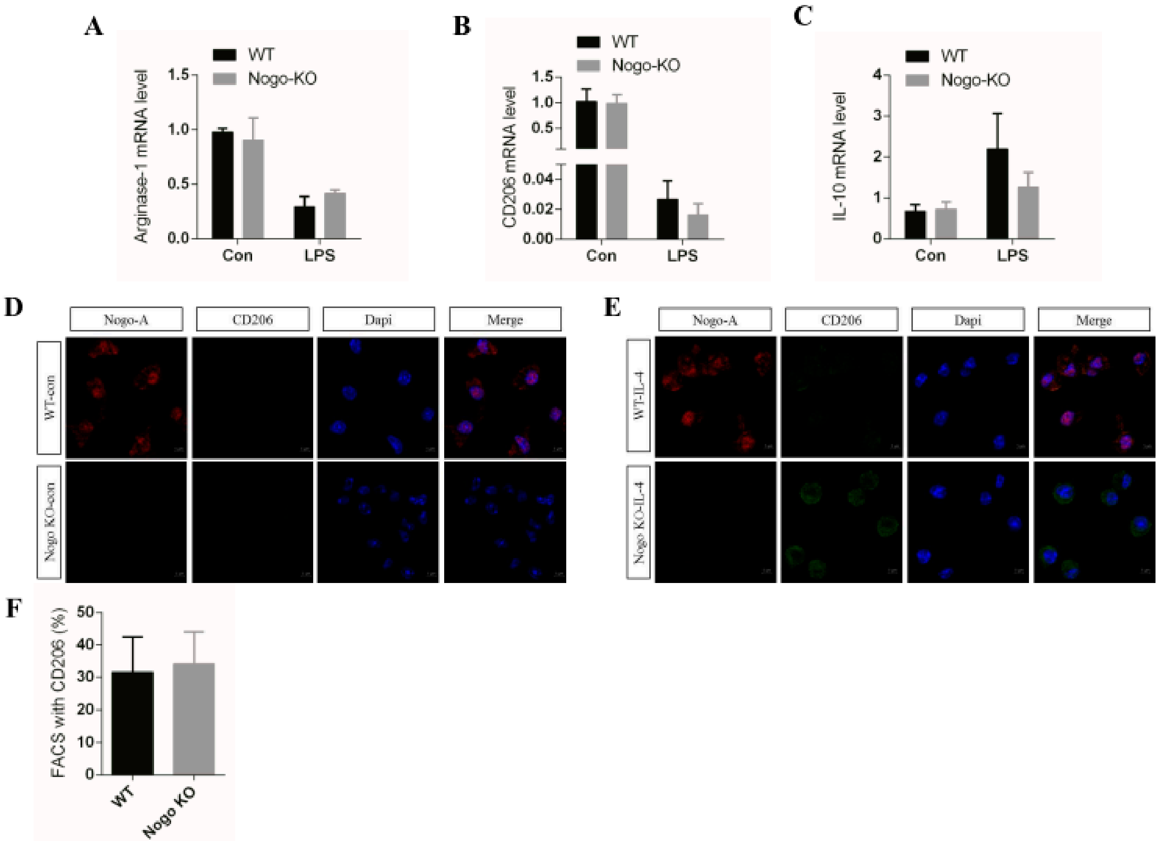

Figure S2: Expression of anti-inflammatory factors in BMDM. (A-C) mRNA levels of M2 factors including arginase-1, CD206, and IL-10 in WT and Nogo- KO BMDM after LPS treatment (100 ng/mL) for 24 h. (D) IF analysis of Nogo-A and the M2 macrophage marker CD206 in WT and Nogo-KO BMDM. (E) IF analysis of Nogo-A and CD206 in WT and Nogo-KO BMDM after IL-4 treatment (20 ng/mL) for 24 h. Nogo-KO BMDM exhibit increased expression of CD206 compared with IL-4-treated WT BMDM. (F) FACS analysis of CD206 in WT BMDM and Nogo-KO BMDM after IL-4 treatment. BMDM were isolated from WT and Nogo-KO mice and cultured for seven days. qPCR results show that gene expression was normalized to that of GAPDH. The results are presented as the mean ± standard error of the mean (n = 3). Statistical significance was determined using Student's t-test. Alexa Fluor (AF)-488 (green) and AF-555 (red) were used as secondary antibodies. Scale bar, 5 μm, ×1000 magnification.

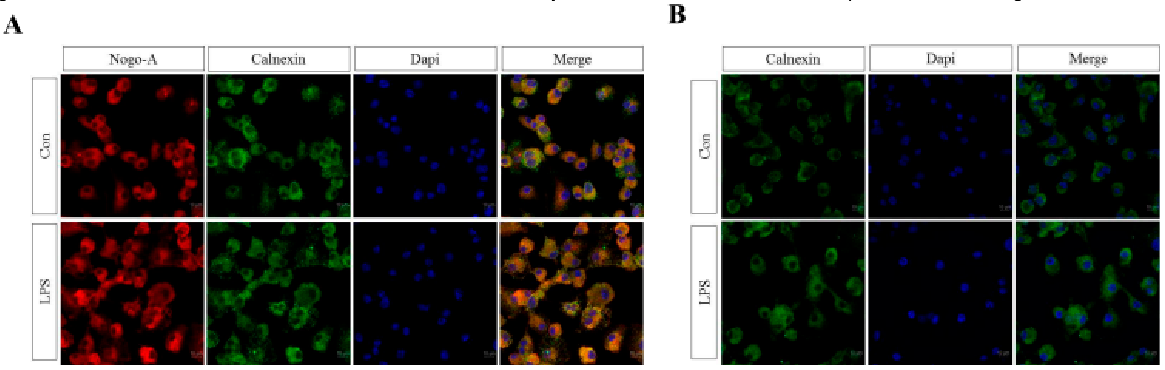

Figure S3: Nogo-A co-localized with calnexin, an endoplasmic reticulum (ER) marker in BMDM. (A) IF co-staining for Nogo-A and calnexin, an endoplasmic reticulum marker, in WT BMDM (n = 3). IF staining showed that Nogo-A and calnexin colocalized in the cytoplasm. (B) IF staining for calnexin in

Nogo-KO BMDM (n = 3). AF-488 (green) and AF-555 (red) were used as secondary antibodies. Scale bar, 10 μm, ×400 magnification.

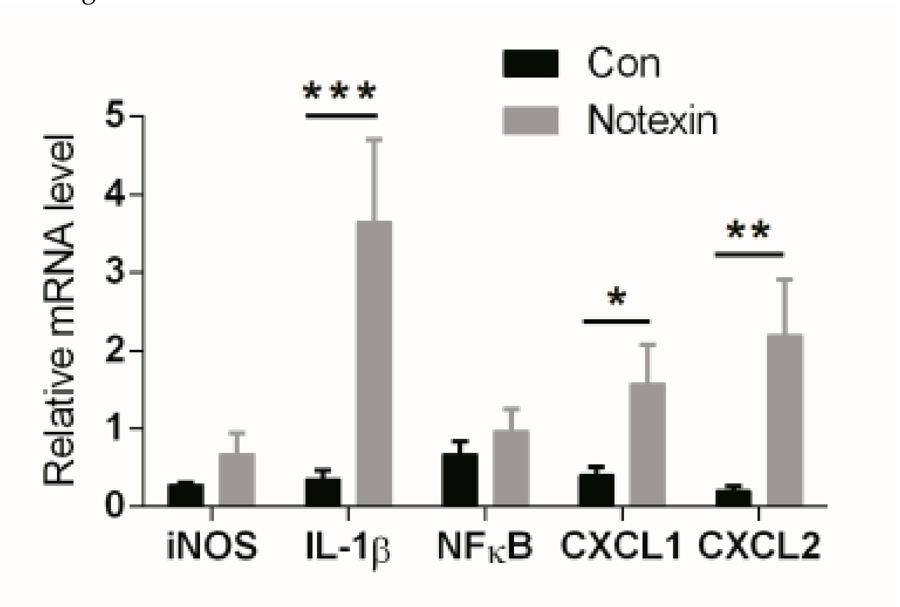

Figure S4: Expression of pro-inflammatory markers in the gastrocnemius muscle from notexin-treated mice. iNOS, IL-1β, NFκB, CXCL1, and CXCL2 are all upregulated in the muscle of notexin- treated mice compared with control mice. Gastrocnemius muscle injury was induced by a single intramuscular injection of 20 μL notexin (12.5 μg/mL) in PBS. All qPCR results show that gene expression was normalized to that of GAPDH. Data are shown as the mean ± standard error of the mean (n = 6). Statistical significance was determined using Student's t-test. Data are denoted by asterisks where * $p < 0.05$, ** $p < 0.01$, *** $p < 0.001$.

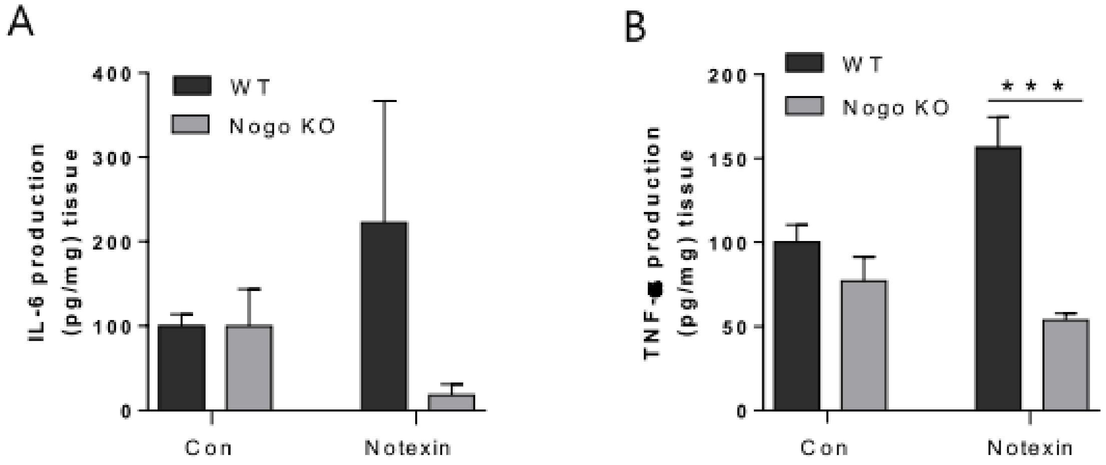

Figure S5: Nogo regulates pro-inflammatory cytokines in notexin induced muscle damage in mice. (A-B) pro-inflammatory cytokines (IL-6 and TNF-α) levels were activated in WT and Nogo KO muscle that were injured with notexin (n=3). However, Nogo-KO injured muscles showed lower expression levels of cytokines comparing to WT muscles. Protein was isolated from WT and Nogo-KO control and injured gastrocnemius muscles and cytokines were measured by ELISA kits. Data are shown as the mean ± standard error of the mean. Statistical significance was determined using Student's t-test. Data are denoted by asterisks, where * $p < 0.05$, ** $p < 0.01$, and *** $p < 0.001$.

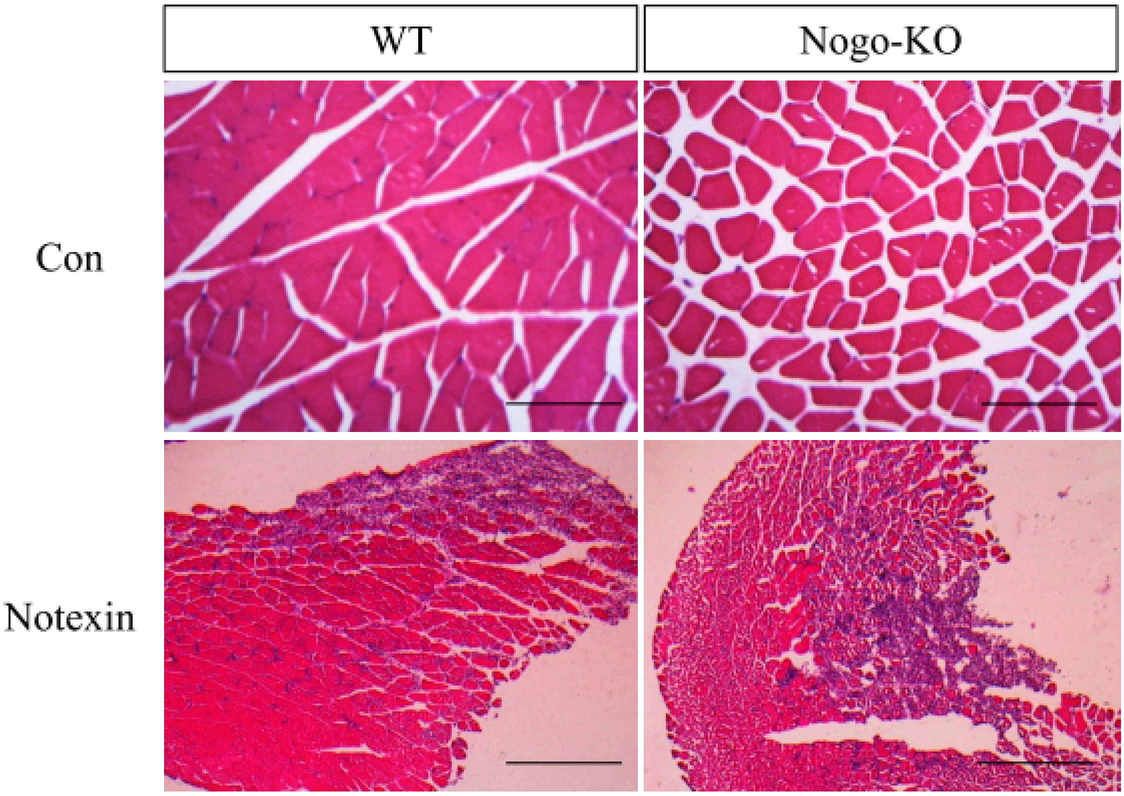

Figure S6: Histological features of control and notexin-treated muscle in WT and Nogo KO mice. Myofibers have appeared normal in control WT and Nogo KO mice in H&E staining. Injured myofibers with infiltration of inflammatory cells have appeared three days post notexin (12.5 μg/mL, 20 μL), a single intramuscular injection in WT and Nogo KO mice in H&E staining. Scale bar represents 200 μm in control and 500 μm in notexin treated groups respectively.
